# Common beans and cowpeas as complementary foods to reduce environmental enteric dysfunction and stunting in Malawian children: study protocol for two randomized controlled trials

**DOI:** 10.1186/s13063-015-1027-0

**Published:** 2015-11-14

**Authors:** Indi Trehan, Nicole S. Benzoni, Alfred Z. Wang, Lucy B. Bollinger, Theresa N. Ngoma, Ulemu K. Chimimba, Kevin B. Stephenson, Sophia E. Agapova, Kenneth M. Maleta, Mark J. Manary

**Affiliations:** Department of Pediatrics, Washington University in St. Louis, One Children’s Place, Campus Box 8116, Saint Louis, MO 63110 USA; Department of Paediatrics and Child Health, University of Malawi, Blantyre, Malawi; University of Texas Southwestern Medical School, Dallas, TX USA; Department of Food Science and Technology, Lilongwe University of Agriculture and Natural Resources, Lilongwe, Malawi; Columbia University College of Physicians and Surgeons, New York, NY USA; Department of Community Health, University of Malawi, Blantyre, Malawi; Children’s Nutrition Research Center, Baylor College of Medicine, Houston, TX USA

**Keywords:** common beans, complementary foods, cowpeas, dual-sugar absorption test, environmental enteric dysfunction, environmental enteropathy, legumes, malnutrition, microbiome, stunting

## Abstract

**Background:**

Interventions to decrease the burden of childhood malnutrition are urgently needed, as millions of children die annually owing to undernutrition and hundreds of millions more are left cognitively and physically stunted. Environmental enteric dysfunction (EED), a pervasive chronic subclinical inflammatory condition among children that develops when complementary foods are introduced, places them at high risk of stunting, malabsorption, and poor oral vaccine efficacy. Improved interventions to reduce the burden of EED and stunting are expected to markedly improve the nutritional status and survival of children throughout resource-limited settings.

**Methods/Design:**

We will conduct, in parallel, two prospective randomized controlled clinical trials to determine whether common beans or cowpeas improve growth, ameliorate EED, and alter the intestinal microbiome during a high-risk period in the lives of rural Malawian children. Study 1 will enroll children at 6 months of age and randomize them to receive common beans, cowpeas, or a standard complementary food for 6 months. Anthropometry will be compared among the three groups; EED will be assessed using a dual-sugar absorption test and by quantifying human intestinal mRNA for inflammatory messages; and the intestinal microbiota will be characterized by deep sequencing of fecal DNA, to enumerate host microbial populations and their metabolic capacity. Study 2 will enroll children 12–23 months old and follow them for 12 months, with similar interventions and assessments as Study 1.

**Discussion:**

By amalgamating the power of rigorous clinical trials and advanced biological analysis, we aim to elucidate the potential of two grain legumes to reduce stunting and EED in a high-risk population. Legumes have potential as an affordable and effective complementary food intervention, given their cultural acceptability, nutritional content, and agricultural feasibility in sub-Saharan Africa.

**Trial registration:**

Clinicaltrials.gov NCT02472262 and NCT02472301.

## Background

Approximately 45 % of all deaths worldwide among children under the age of five, that is, 3.1 million deaths annually, are directly or indirectly related to undernutrition [1]. Additionally, stunting permanently affects an additional 165 million children worldwide, and reduces the affected individual’s physical, immunological, and cognitive capacity throughout his or her lifetime. Stunting is estimated to account for 21 % of all disability-adjusted life years in children. Both stunting and wasting are causally related to the dietary intake and gut health in children younger than 3 years.

### Environmental enteric dysfunction

In developing, impoverished settings, a common gut inflammatory condition known as environmental enteric dysfunction (EED, formerly known as environmental enteropathy, and even earlier as tropical enteropathy) develops early in life [2, 3]. While subclinical, EED predisposes children to more clinically manifest forms of malnutrition: wasting and stunting. Environmental enteric dysfunction is characterized by T-cell infiltration of the intestinal mucosa, leading to a chronic inflammatory state with increased intestinal permeability, translocation of gut microbes, micro- and macronutrient malabsorption, poor weight gain, stunted physical and cognitive development, frequent enteric infections, and decreased response to enteric vaccines [4]. While a precise etiology of EED has not been identified, it is epidemiologically associated with unsanitary living conditions [5]. Given the significant contribution of malnutrition to childhood morbidity and mortality, meaningful progress on reducing EED might establish a lasting foundation for progress against global hunger [6, 7].

Environmental enteric dysfunction develops within the first three years of life, a high-risk period marked also by the transitions from exclusive breastfeeding to mixed feeding with complementary foods and then to complete reliance on adult foods for sustenance [6]. In traditional sub-Saharan African societies, complementary foods are dominated by monotonous, protein-poor, micronutrient-poor starches, such as maize, cassava, and sorghum. Alternative, yet culturally acceptable, complementary foods that can provide a better balance of nutrients might decrease EED and improve growth.

### Legumes

Legumes provide such an opportunity, as their protein content is significantly higher than cereals, and they are rich in dietary fiber, starch, minerals, vitamins, and antioxidants. Common beans and cowpeas, for example, have 3–4-fold more protein per gram than corn. The zinc content in legumes is also relatively high and might further decrease the progression of EED, as we have demonstrated recently in a prospective randomized trial [8]. Legumes make an excellent complementary food for children weaning from exclusive breastfeeding and, with appropriate preparation, are quite digestible and well-tolerated [9]. Successful legume–maize blends have, in fact, already been developed in the past and demonstrated favorable acceptability profiles in children younger than one year; they were also nutritionally sound as a weaning supplement [10].

Additionally, interventions with anti-inflammatory effects might improve gut health, since EED is a chronic inflammatory condition, both in the lamina propria of the intestinal tract and systemically, owing to translocation of enteric bacteria and their products across the compromised mucosal border [11]. Simply attacking the bacterial confounders of EED alone is unlikely to be sufficient, given past failures to improve EED via the use of probiotics [12] or antibiotics [13]. A growing body of evidence [14–17] suggests that a diet enriched in legumes decreases markers of inflammation and is correlated with decreases in illnesses with inflammatory components such as colorectal cancer and cardiovascular disease [18].

Evidence from molecular and animal models supports a mechanistic explanation whereby certain legumes may have direct anti-inflammatory effects on the intestines of children with EED. For example, the indigestible portion of common beans downregulates signaling pathways that lead to inflammation in a mouse model of colon cancer [19]. Intriguingly, there is probably an essential microbial component to the downregulation of this inflammatory cascade: when fermented by normal gut flora, the indigestible fraction from common bean produces short-chain fatty acids that inhibit colon cancer HT-29 cell growth and modulates protein expression associated with apoptosis, cell cycle arrest, and proliferation. These effects were also demonstrated morphologically [20]. The indigestible fraction of common beans also protects against chemically induced crypt inflammation in the rat colon [21, 22].

Cowpeas (*Vigna unguiculata*) are also attractive for study, as they grow well in Africa, are culturally accepted, and are a hardy, drought-tolerant, crop. Cowpeas also have significant anti-inflammatory effects, mediated by specific phenolic profiles and antioxidant activity [23]. These phenolic compounds are also active after cooking and simulated enzymatic digestion [24].

### Microbiome

The evolving intestinal microbiome in African children also warrants study. Although no specific microbial populations or disruptions have yet been linked to EED [25], evidence suggests that a disruption in the relative populations among the four dominant bacterial phyla (*Firmicutes*, *Bacteroidetes*, *Actinobacteria*, and *Proteobacteria*) are correlated with gut mucosal breakdown in inflammatory bowel disease [26, 27]. We have demonstrated recently that empiric antimicrobial interventions in severely malnourished children improve nutritional recovery [28], and that specific disturbances in the maturation of young children’s intestinal microbiota are linked to severe malnutrition [29]. Hence, it would be instructive to understand the effect that specific food-based interventions have on commensal gut organisms and their metabolic capacity [30]. A recent study comparing the gut microbiota in children from rural Burkina Faso, who consumed a diet rich in legumes, with European children showed a relative lack of potentially pathogenic *Enterobacteriaceae* in the African children, conceivably protecting these children from severe gut inflammation and bacterial translocation [31].

### Study goals

In our previous work, we have found EED in more than 80 % of children studied in rural Malawi [8, 12, 13, 32]. In these studies, we plan to investigate the effects on childhood growth, gut health, and the intestinal microbiome caused by the systematic introduction of cowpeas and common beans into the complementary diet of vulnerable Malawian children.

## Methods/Design

### Study design

**Fig. 1:**
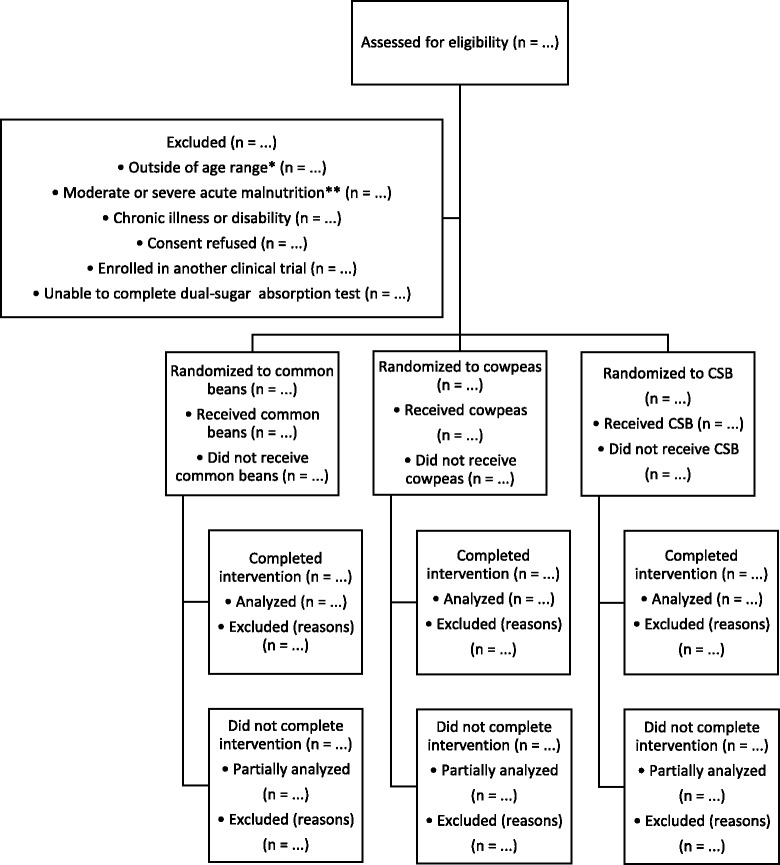
Patient flow diagram for each of two clinical trials *Children in Study 1 will be enrolled at 5.5–6.5 months of age and followed for 6 months. Children in Study 2 will be enrolled at 12–23 months of age and followed for 12 months. **Defined as having a weight-for-height Z score < −2, a mid-upper-arm circumference < 12.5 cm, or bilateral pitting edema suggestive of kwashiorkor. CSB; corn–soy blend

In parallel, we will conduct two prospective, randomized, investigator-blinded, controlled trials to compare the effectiveness of legume consumption with the customary local complementary food on infant and toddler growth and gut health in rural Malawi. Study 1 will enroll children at 5.5–6.5 months of age and provide the randomized intervention for 6 months. Study 2 will enroll children at 12–23 months of age and provide the randomized intervention for 12 months. A probable patient flow diagram for each trial is shown in Fig. 1.

### Study setting

The studies will enroll children in the village clusters surrounding Limela, Machinga District, and Masenjere, Nsanje District, Malawi. These villages are populated primarily by subsistence farmers who grow and consume maize as their primary staple crop. Water is generally acquired several times per day from boreholes or nearby streams. Most homes are quite small and are constructed from mud bricks and have thatch, or occasionally sheet metal, roofs. Defecation generally occurs outdoors or in small pit latrines. Children have frequent contact with farm animals, such as chickens and goats. Electricity, running water, and other utilities are invariably absent in homes. Almost all household chores and child-rearing is performed by mothers or other female figures; in these villages, most of the women are illiterate and it is quite rare for women to have completed primary school.

### Ethical considerations

We aim to complete the study using the highest ethical standards for clinical trials research, in accordance with the Declaration of Helsinki [33]. Ethical approval has been obtained from the University of Malawi College of Medicine Research and Ethics Committee and from the Human Research Protection Office at Washington University in St. Louis. Letters of support have also been obtained from the district health officers in the two districts where the study will be conducted. Local village chiefs surrounding the study sites have also given their consent and support for the studies. Each child’s caregiver will be briefed on the study procedures and interventions. Verbal and written informed consent will be obtained from all caregivers. Caregivers who are unable to sign or write their names will be asked to document consent using their thumbprints.

### Eligibility criteria

Children living within walking distance of one of the two study sites and within the enrollment ages for the two studies (5.5–6.5 months for Study 1 or 12–23 months for Study 2) will be screened for eligibility. Children without severe or moderate acute malnutrition (defined as a weight-for-height Z score < −2, mid-upper arm circumference < 12.5, or pitting edema suggestive of kwashiorkor) and without evidence of congenital or other chronic diseases will be considered eligible. Additional exclusion criteria will include current treatment for acute malnutrition and any report from the caregiver that the child currently has diarrhea. Caregivers who report that they will be moving away from the study area during the course of the study (6 months for Study 1 or 12 months for Study 2) will also be excluded.

### Primary outcome measures and sample size

The primary outcome measures of interest will be change in height-for-age Z score and improvement in the major biomarker of environmental enteropathy, the lactulose to mannitol ratio (L:M) [34]. To detect a difference in change in length of 1.1 cm, which corresponds to a change in height-for-age Z score of 0.45 units at 12 months of age, 79 children are needed in each group. This sample size was calculated for a two-tailed test using G*Power 3.1.9.2 [35] at a significance level of 0.05 and 80 % power.

We also desire to detect a (medium) effect size of 0.5 in the L:M test after the legume intervention, again with 80 % power at a significance level of 0.05. Given these parameters, if the L:M results were to follow a normal distribution, thereby allowing the use of Student’s *t* test to compare the differences between independent means, 64 children would be needed in each group. If the L:M results are not normally distributed and require nonparametric analysis with the Wilcoxon–Mann–Whitney test, then up to 74 children might be needed per group. In recent studies, we have experienced approximately a 5 % rate of insufficient urine specimens [8]. We therefore very conservatively estimate that this rate might increase to 15–20 %. Thus, we believe that enrolling 300 children with a 15–20 % rate of failure to obtain sufficient urine (owing to dropouts and specimen collection failures) will yield complete analysis for at least 240 children, or 80 in each group.

Should 100 children indeed be enrolled and retained in each intervention arm, the power achieved for the height-for-age Z score criterion is 89 % and for the L:M criteria is 94 % (if normally distributed) and 93 % (if non-normally distributied).

### Intervention groups

Children in each study will be randomly assigned to receive common bean flour, cowpea flour, or corn–soy blended flour in a 1:1:1 ratio across both study sites. The legume flours will be produced from locally grown and harvested legumes available in local markets, after sorting to remove rotten beans and any other foreign materials. After trialing several different methods of cooking the legumes in small acceptability tests with local children, we determined that roasting and milling the beans into a fine flour to be added to traditional corn porridge meals was the most palatable and practical method for introducing these new foods to the diet. Corn–soy blended flour will be purchased from local suppliers and caregivers will also be taught to administer this control intervention in traditional porridge.

Children in Study 1 will receive approximately 40 % of their daily energy requirements from complementary feeding (200 kcal/day at 6–8 months of age; 300 kcal/day at 9–11 months of age) [36] via the intervention foods (Table 1). Given that the duration and amount of breastfeeding can be so variable in children after their first year of life, children in Study 2 will receive approximately 15–20 % of the estimated total daily energy requirement for nonbreastfed children [37, 38] via the intervention foods (Table 2).

**Table 1:** Quantities and calories of flours to be consumed by subjects enrolled in Study 1

Age group	Common beans or cowpeas (kcal/day)	Legume flour (g/day)	Number of 18 ml scoops/day	Total amount (kg) per 6-week food distribution
6–8 months	80	21	2	0.88
9–11 months	120	31.5	3	1.32
Age group	Corn–soy blend (kcal/day)	Corn–soy blended flour (g/day)	Number of 18 ml scoops/day	Total amount (kg) per 6 week food distribution
6–8 months	76	20	2	0.84
9–11 months	114	30	3	1.26

**Table 2:** Quantities and calories of flours to be consumed be subjects enrolled in Study 2

Age group	Common beans or cowpeas (kcal/day)	Legume flour (g/day)	Number of 18 ml scoops/day	Total amount (kg) per 6-week food distribution
12–23 months	160	42	4	1.76
24–35 months	200	52.5	5	2.21
Age group	Corn–soy blend (kcal/day)	Corn–soy blend flour (g/day)	Number of 18 ml scoops/day	Total amount (kg) per 6 week food distribution
12–23 months	152	40	4	1.68
24–35 months	190	50	5	2.1

### Choice of legume intervention

The specific intervention legumes thus chosen were Andean white common beans (*Phaseolus vulgaris*) and cowpeas (*Vigna unguiculata*). The rationale for this choice was based on several considerations. We felt that the legumes chosen must be able to be grown widely by subsistence farmers in sub-Saharan Africa (that is, the families of children with EED) [39]. Common beans and cowpeas met this criteria, as they are the most commonly grown legumes in Africa. Cowpeas, for example, grow well in marginal soils that are 85 % sand and less than 1 % organic matter, as is often found in Africa. As legumes, both also fix nitrogen in the soil rather than extracting it.

These two legumes are also good choices since their nutrient content adds diversity and quality to the habitual African diet, most notably in terms of protein content. Of course, the legumes chosen must not pose any significant health risks; common beans do pose a minor risk with phytohemagglutinin, but are otherwise considered to be very safe. White common beans were selected in part because their phytohemagglutinin content is one-third that of red common beans [40]. The legumes chosen also support microbiota that produce short-chain fatty acids or microbiota that are associated with an anti-inflammatory effect [41, 42].

### Preparation of legume flours for consumption

After hand-sorting of the common beans and cowpeas to remove rotten beans and those infected with weevils, beans will be roasted in 100 kg batches to an internal temperature of 120–130 °C for 45–50 minutes. This serves several purposes: (1) to improve palatability; (2) to decrease the risk of microbial contamination; and (3) to decrease the risk of phytohemagglutinin poisoning by denaturing the toxin found in raw beans. Roasted beans are then milled into a flour.

Caregivers of children enrolled in the study will be taught to prepare the flours by mixing the allotted amount with a small amount of unheated water and mix with the traditional maize porridge (‘phalla’) that is the traditional staple complementary food for children of this age. This porridge is traditionally prepared by adding maize flour to a pot of boiling water and stirring continuously for 10–15 minutes until the desired consistency is obtained. By not boiling the intervention flour, the quantities of nutrients and fibers in the flours will be unchanged from the amounts measured after production of the flours. We anticipate that this simple preparation method will also improve compliance, as caregivers do not need to cook anything extra for this one child – instead, a small portion of porridge can be set aside and the assigned intervention flour with water can be added to the already cooked maize porridge.

### Randomization methods and blinding

Block randomization will be employed, using a computer-generated list in permuted blocks of nine. Allocation groups will be pre-printed on paper that will be sealed in opaque envelopes. The caregivers will then each draw out one opaque envelope, which will be opened in their presence, to determine their intervention groups.

Each type of flour will be packaged identically, except for the color of the lid used on the jars that contain the interventions. The allocation code connecting the lid colors to the types of flour is known only to the individuals involved in the packaging of the flours. All field research nurses, investigators, and laboratory personnel will be kept blinded to the color code as well as to each child’s assigned color throughout the study. Caregivers will not be told which type of flour they have been assigned, but while the flours have a very similar appearance and texture, it is reasonable to assume that caregivers may deduce the type of flour their children has been assigned by tasting or smelling the flour themselves. To reflect this possibility, we thus consider these to be investigator-blinded studies.

### Study participation

After obtaining verbal and written consent from caregivers of eligible children, randomization will occur and basic sociodemographic, food frequency, and economic data will be collected. Recent illnesses during the previous 7 days will be queried at each visit [43]. At the initial visit, anthropometric data will be gathered to ensure that the child is not acutely malnourished and thus ineligible for the study. If eligible, the child will continue with the dual-sugar absorption test and stool collection. After successful completion of the dual-sugar absorption test and stool collection, a 6-week supply of the assigned flour intervention will be provided and the caregiver will be educated on how to prepare and feed the flour to the child. The complete schedule of study visits, testing, and distribution of flours is provided in Table 3 for Study 1 and Table 4 for Study 2.

**Table 3:** Study participation schedule for subjects enrolled in Study 1

	Anthropometry	Dual-sugar absorption test	Stool collection for EED biomarker and microbiome studies	Intervention flour distribution
Week 0	X	X	X	X
Week 2	X		X	
Week 6	X		X	X
Week 12	X	X	X	X
Week 18	X		X	X
Week 24	X	X	X

**Table 4:** Study participation schedule for subjects enrolled in Study 2

	Anthropometry	Dual-sugar absorption test	Stool collection for EED biomarker and microbiome studies	Intervention flour distribution
Week 0	X	X	X	X
Week 2	X		X	
Week 6	X		X	X
Week 12	X	X	X	X
Week 18	X			X
Week 24	X	X	X	X
Week 30	X			X
Week 36	X	X	X	X
Week 42	X			X
Week 48	X	X	X	

### Anthropometry

Weight will be assessed to the nearest 5 g using standard digital scales (seca 334, Chino, CA, USA) and height will be measured to the nearest 0.1 cm using a rigid height board (seca 417, Chino, CA, USA). Mid-upper-arm circumference will be measured on each child’s left upper arm using standard flexible plastic insert tapes to the nearest 0.1 cm (TALC, St. Albans, UK). Each child will also be assessed for kwashiorkor by pressing with the pad of the thumb on the dorsum of the foot and observing for pitting edema. Any child with a weight-for-height Z score less than −2, a mid-upper arm circumference less than 12.5, or pitting edema will be classified as acutely malnourished [44]. Malnourished children will be excluded from enrollment in the study and will instead be treated with ready-to-use lipid-nutrient spreads according to usual protocols until they recover [45].

### Dual-sugar absorption test

The dual-sugar permeability test [34] will be used as a standard noninvasive measure of gut integrity, as it correlates with the severity of small bowel histological changes [46], severity of diarrhea [47], micronutrient status [48, 49], and growth in weight and length [50]. The ratio of urinary excretion of lactulose to mannitol is used to measure intestinal mucosal permeability with higher ratios indicative of increased intestinal permeability.

To participate in the test, children will be asked to take no food (water and breast milk are permitted) after 10 p.m. on the previous night. The test begins when each child drinks a 20 ml solution containing 1 g mannitol and 5 g lactulose. Children will be carefully observed by research assistants to ensure complete ingestion of this solution; any child who spits up, vomits, or refuses the solution will be brought back for testing on another day. Adhesive urine bags will then be placed on the child’s perineum and monitored for urine output. As soon as urine is noted in the bag, it will be removed and the urine transferred to a clean container containing 10 mg merthiolate to prevent bacterial degradation of the excreted sugars, and a new bag will be placed. After 3–3.5 hours, children will be given water to drink to facilitate the collection of urine. After the child voids for the first time after 4 hours, the urine collection will be omplete. The total amount of urine voided during the study will then be quantified and an aliquot flash frozen for analysis.

The concentrations of lactulose and mannitol in the total voided urine will be determined using HPLC, as described previously [51]. Given a recent report that HPLC is less reliable than liquid chromatography-tandem mass spectrometry [52], we previously compared our L:M results by HPLC on 115 samples with those obtained by the Mayo Clinic performed using liquid chromatography-tandem mass spectrometry and we found the results to be highly correlated (data not shown). The concentrations of these sugars in the urine will then be multiplied by the total amount of urine voided to provide a measure of the total amount of these sugars voided. The ratio of the sugars voided in the urine, as well as the amount voided as a percentage of the amount ingested, will be computed and compared between groups.

### Exploratory analysis of EED biomarkers in fecal samples

Stool samples will be collected using plastic-lined diapers and aliquots of stool transferred using sterile metal spatulas into cryovials and flash frozen in liquid nitrogen within five minutes of defecation. Novel fecal biomarkers will be sought to assess bowel integrity and levels of inflammation noninvasively. Evaluation of these markers of barrier dysfunction might pave the way for assessment of intestinal mucosal permeability and inflammatory response in diagnosing the degree and progression of EED in vulnerable populations without the need for the relatively lengthy and cumbersome dual-sugar absorption test [53–61]. Markers of interest include cytokines, interleukins, immunoglobulins, REG1A, REG1B, calprotectin, and zonulin.

We also plan to continue our exploration of human mRNA transcripts of putative inflammatory and antimicrobial mediators that are upregulated in EED and found in fecal samples. Nucleic acids are first extracted using a NucliSens EasyMAG kit (BioMérieux), which is a bead-based RNA-specific method. A digital droplet PCR is used to detect low copy numbers of human mRNA reliably [62]. The digital droplet PCR is unique in that after the PCR is set up in the aqueous phase, the reaction is emulsified in oil, creating 20,000 aqueous bubbles that are counted by laser for positive fluorescence of the probe of interest. The quantities of mRNA in a given sample are normalized to a housekeeping gene present in abundant quantities. A number of promising targets that correlate with EED have already been identified (unpublished data) and these will be pursued further in this study, with the aim of identifying one or more that may serve as less cumbersome biomarkers of EED. Newer whole human transcriptome microarray technology [63, 64] will also be employed to interrogate these samples, as another approach to identify these biomarkers.

### Microbiome analysis

To investigate correlations between bacterial populations in the gut and EED, as well as the effects of the legume interventions on gut microbe populations, bacterial genomic DNA will be prepared separately from fecal samples obtained from study children. To prepare the stool samples for sequencing, genomic DNA will be extracted using standard protocols (http://hmpdacc.org/resources/tools_protocols.php) V1-V3 variable regions of bacterial 16S rRNA genes will be amplified by PCR and the resulting amplicons will be subjected to high throughput sequencing. We will use the to Illumina Miseq platform for targeted 16S rRNA sequencing. This deep sequencing permits the recognition of rare members of the microbiota that may be shared or that may discriminate these human populations. 16S rRNA sequences will be processed through the pipeline developed by human microbiome projects at The Genome Institute at Washington University. In brief, quality trimming, denosing and chimera removal will be performed to yield high quality reads [65]. Reads passing the above filtering steps will be classified from the phylum to the genus level using an automated implementation of the Ribosomal Database Project’s Classifier [66]. Species-level bacterial phylotypes will be defined as organisms sharing at least 97 % nucleotide sequence identity in the V1-V3 regions of their 16S rRNA genes using the Mothur software package [67].

Shotgun sequencing will be performed to examine the taxa that are prominently represented in the infant microbiome but inefficiently captured by PCR primers designed to target other variable regions. Whole genome sequencing libraries will be prepared following a standard protocol from Illumina. Ten GB 100 bp pair-end reads per sample will be sequenced on the Illumina HiSeq platform. Reads passing quality filtering and human contamination removal will be classified to species level based on nucleotide sequence alignment to reference genomes using Real Time Genomics map software (Real Time Genomics, San Bruno, CA, USA). Read mapping results will be transformed to values representing the coverage of each reference genome using refcov software (http://gmt.genome.wustl.edu/packages/refcov/index.html). The coverage values are then scaled to a million aligned reads to calculate ‘depth of coverage per million reads’ values. Taxa-based analysis will be the same as the 16S rRNA genes approach described above.

### Data management and analysis

Clinical data such as anthropometry, demography, and morbidity data, will be collected on standardized forms by field workers. Field workers will be trained and validated with the questionnaires and measurements they collect prior to data collection. Anthropometric Z-scores will be calculated from the 2006 WHO Multicentre Growth Reference Survey [68]. Data will be double-entered into a password-protected Microsoft Access database by research assistants blinded to food assignments. All discrepancies will be resolved by examination of the original data cards and discussion with the relevant field workers, possibly including repeat examination of the child and questioning of the caregiver as needed. Once all values are entered and discrepancies resolved, the data set will be locked.

Comparisons will be made between cowpea flour and corn–soy blended flour, separately from common beans and corn–soy blend. Fisher’s exact test will generally be used to compare discrete parameters, and Student’s *t* test will be used for continuous parameters. A difference with *P* < 0.05 will be considered statistically significant. These statistical methods will also be used for all clinical data and EED measures (*e.g.*, L:M ratio), which are continuous measures of EED status.

The microbiome data analyses and interpretation require specialized tools. Read-based metabolic profiling of the microbial communities is performed by first using whole genome sequencing reads from each sample to probe the *Kyoto Encyclopedia of Genes and Genomes* using Mblastx (MultiCoreWare, St. Louis, MO) and then search results are run through the metabolic pathway pipeline developed as part of the Human Microbiome Project to obtain enzyme and pathway abundance and coverage from metagenomic communities. Differentially expressed enzymes and pathways between groups (both between intervention groups as well as within the same individual, for example before and after intervention) are identified using LEfSe (http://huttenhower.sph.harvard.edu/lefse). Principal component analysis will be performed to identify cluster patterns based on the gene content and metabolic pathway abundance between the legume and corn–soy blend groups.

All microbiome data will be rarefied to ensure the sample comparisons are at the same read depth. Ordination methods will facilitate the pattern analysis of the high dimensional microbiome data by reducing its dimensionality to 2–3 dimensions, such that the original distance between the samples is preserved to a large degree. In particular, principal component analysis with Bray–Curtis dissimilarity will be used to identify the sample cluster pattern based on the microbial community structures.

A robust statistical approach that is widely used in ecology and metagenomics, Permutational Multivariate Analysis of Variance (perMANOVA in the R software package, http://www.r-project.org), will be used to test the significance of differences at the whole bacterial community level between two or more groups. Dissimilarity matrices will be generated based on the taxon abundance table, and will be treated as the dependent variable in the model. We will use metastats, a program developed in the Human Microbiome Project, to detect differentially abundant taxa between groups [69]. The statistical tests will be corrected for false discovery rates using the q-values approach, an approach that has been effectively applied in metagenomic studies for identifying the taxa of interest. Indices such as richness (the total different number of taxa) and Shannon diversity (the number of taxa present as well as the relative abundances) that categorize the complexity of the bacteria community will be computed. Student’s t-test will be used to compare the diversity differences between groups.

## Discussion

Childhood undernutrition remains a formidable challenge in global health, contributing greatly to the continued unacceptably high rates of mortality among young children, and even more so to decreased achievement of full adult cognitive and physical growth potential [70]. No significant reduction in the overall damage caused by undernutrition is likely to be successful without making major inroads against EED and stunting [4, 71], especially since the two have a synergistic detrimental effect [72, 73]. We have presented here the outlines of two randomized investigator-blinded prospective controlled clinical trials to test the hypothesis that dietary supplementation with legumes at times of peak vulnerability in child growth and development will decrease EED and stunting.

Prior studies using pharmacological interventions to reduce EED have only shown relatively modest and transient improvements in gut health and it remains unclear whether any such intervention targeting inflammation will be effective in reducing EED [74]. Indeed it is quite possible that sanitation and hygiene interventions may be the most effective interventions to ameliorate EED and stunting [5, 75], but the extremely high cost and logistical challenges of implementing such interventions throughout the developing world unfortunately remain exceedingly high.

The trials presented here are novel in that we have taken a food-based approach to targeting EED [11], carefully selecting two legumes – common beans and cowpeas – that have high levels of both macronutrients and micronutrients that make them appealing choices as complementary foods. We also postulate that their anti-inflammatory effects in the gut may also help to decrease the chronic inflammatory state that accompanies EED [73] and stunting [76].

Beans have been cultivated for more than 700 years and were harvested in Africa long before the colonial era. They might thus serve as major sources of protein in southeast Africa if proven to be of benefit in these young children. Indeed, the Great Lakes region of Africa has the highest per capita bean consumption in the world, demonstrating the cultural acceptability of such legumes in our target population [77]. However, the consumption of legumes among young children in rural Malawi remains extremely low due to the preference for maize and other carbohydrate-rich staples [78]. Legumes could therefore serve as a complementary food in this high-risk population, with key measurable endpoints and biomarkers, including markers of EED and growth parameters.

One limitation of the study is the lack of complete blinding of the assigned flour interventions between the three groups. Although the different flours will be packaged similarly and, at first pass, have very similar appearances and textures, a curious caregiver could ultimately determine the nature of the intervention by taste and/or smell, and subsequently trade their assigned intervention with another participant from the same village. We are optimistic that this will be a relatively unlikely occurrence, as our past research into this possibility in a very similar population did not demonstrate any evidence of sharing or trading either assigned food [79] or pharmacological [28] interventions. Extensive education of caregivers of trial participants (virtually all of whom will be the household matriarch) and community engagement with village leaders about the need to comply with the assigned interventions should also decrease this possibility. Our preliminary data from acceptability studies did not demonstrate that the children themselves did not like the flours, and thus they will not likely be the motivation for any trading either.

The possibility of sharing of the flours with other members in the household also exists, but this should be relatively even across the three different intervention arms, and relatively unlikely since the amount of flour to be consumed each day is rather small and unlikely to be particularly satisfying to a hungry family member.

As the trials get underway, we may also use this robust study design and multi-site enrollment of two unique age groups with a presumed range of pathology to consider the addition of sub-studies to explore concepts such as household food consumption, cognitive and physical development, serum or other functional biomarkers of nutritional status, and other health variables such as acute infectious symptoms.

## Trial status

Recruitment of trial participants began in July 2015. Interaction with study subjects is expected to last approximately 18 months.

## Abbreviations

EED: environmental enteric dysfunction
HPLC: high-performance liquid chromatography
L:M: lactulose to mannitol ratio
PCR: polymerase chain reaction
